# Long-term effectiveness and cost-effectiveness of high versus low-to-moderate intensity resistance and endurance exercise interventions among cancer survivors

**DOI:** 10.1007/s11764-018-0681-0

**Published:** 2018-03-01

**Authors:** C. S. Kampshoff, J. M. van Dongen, W. van Mechelen, G. Schep, A. Vreugdenhil, J. W. R. Twisk, J. E. Bosmans, J. Brug, M. J. M. Chinapaw, Laurien M. Buffart

**Affiliations:** 1 Department of Public & Occupational Health, and the Amsterdam Public Health research institute, VU University Medical Center, Amsterdam, The Netherlands; 2 Department of Health Sciences and the Amsterdam Public Health research institute, Faculty of Earth and Life Sciences, Vrije Universiteit Amsterdam, Amsterdam, The Netherlands; 3 Department of Sports Medicine, Máxima Medical Center, Veldhoven, The Netherlands; 4 Máxima Oncology Center, Eindhoven and Veldhoven, The Netherlands; 5 Department Medical Oncology, Maastricht University Medical Center, Maastricht, The Netherlands; 6 Department of Epidemiology and Biostatistics, and the Amsterdam Public Health research institute, VU University Medical Center, De Boelelaan 1089a, 1081 HV Amsterdam, The Netherlands; 7 Department of Epidemiology and Biostatistics, and the Amsterdam Public Health research institute, VU University Medical Center, De Boelelaan 1089a, 1081 HV Amsterdam, The Netherlands; 8 Amsterdam School of Communication Research (ASCoR), University of Amsterdam, Amsterdam, The Netherlands

**Keywords:** Exercise intensity, Neoplasms, Physical fitness, Fatigue, Quality of life, Cost-effectiveness

## Abstract

**Purpose:**

This study aimed to evaluate the long-term effectiveness and cost-effectiveness of high intensity (HI) versus low-to-moderate intensity (LMI) exercise on physical fitness, fatigue, and health-related quality of life (HRQoL) in cancer survivors.

**Methods:**

Two hundred seventy-seven cancer survivors participated in the Resistance and Endurance exercise After ChemoTherapy (REACT) study and were randomized to 12 weeks of HI (*n* = 139) or LMI exercise (*n* = 138) that had similar exercise types, durations, and frequencies, but different intensities. Measurements were performed at baseline (4–6 weeks after primary treatment), and 12 (i.e., short term) and 64 (i.e., longer term) weeks later. Outcomes included cardiorespiratory fitness, muscle strength, self-reported fatigue, HRQoL, quality-adjusted life years (QALYs) and societal costs. Linear mixed models were conducted to study (a) differences in effects between HI and LMI exercise at longer term, (b) within-group changes from short term to longer term, and (c) the cost-effectiveness from a societal perspective.

**Results:**

At longer term, intervention effects on role (*β* = 5.9, 95% CI = 0.5; 11.3) and social functioning (*β* = 5.7, 95%CI = 1.7; 9.6) were larger for HI compared to those for LMI exercise. No significant between-group differences were found for physical fitness and fatigue. Intervention-induced improvements in cardiorespiratory fitness and HRQoL were maintained between weeks 12 and 64, but not for fatigue. From a societal perspective, the probability that HI was cost-effective compared to LMI exercise was 0.91 at 20,000€/QALY and 0.95 at 52,000€/QALY gained, mostly due to significant lower healthcare costs in HI exrcise.

**Conclusions:**

At longer term, we found larger intervention effects on role and social functioning for HI than for LMI exercise. Furthermore, HI exercise was cost-effective with regard to QALYs compared to LMI exercise.

**Trial registration:**

This study is registered at the Netherlands Trial Register [NTR2153 [http://www.trialregister.nl/trialreg/admin/rctview.asp?TC=2153]] on the 5th of January 2010.

**Implications for Cancer Survivors:**

Exercise is recommended to be part of standard cancer care, and HI may be preferred over LMI exercise.

## Introduction

Supervised exercise can contribute to counteracting the negative side effects of cancer and its treatments [1]. Systematic reviews demonstrated safety and beneficial effects of exercise on physical fitness [2], fatigue [3], and health-related quality of life (HRQoL) [4], during and after cancer treatment. However, previous studies predominantly reported short-term effects [4, 5]. The few studies that included a longer-term follow-up (≥ 6 months) showed that the benefits of exercise were maintained for HRQoL [4], but not for fatigue [5]. For other outcomes (e.g., physical fitness), longer-term effects are unclear. Therefore, more research on the longer-term effectiveness of exercise in cancer survivors is warranted.

As resources are scarce, decisions on the implementation of healthcare programs are guided not only by their health effects but also by their additional costs in relation to these effects (i.e., cost-effectiveness). Therefore, it is important that state-of-the-art cost-effectiveness analyses of healthcare programs are performed [6]. Cost-effectiveness analyses of exercise interventions in cancer survivors are scarce [7, 8]. A systematic review compared exercise interventions to usual care in patients with various diseases, including cancer, found acceptable incremental cost-effectiveness ratios or cost savings [7]. Similar results were found by a systematic review evaluating the cost-effectiveness of multidimensional cancer rehabilitation programs [8]. However, despite the fair methodological quality of the reviewed studies, the heterogeneity across interventions hampered solid conclusions about their cost-effectiveness.

The present study reports the effectiveness and cost-effectiveness of the randomized controlled Resistance and Endurance exercise After ChemoTherapy (REACT) study at longer term (i.e., 64 weeks) [9]. At short term (i.e., 12 weeks), high intensity (HI) and low-to-moderate intensity (LMI) exercise interventions significantly improved cardiovascular fitness and HRQoL and reduced fatigue compared to a wait list control (WLC) group, with some indication for a dose-response relationship for exercise intensity on cardiorespiratory fitness [9]. Also, HI and LMI exercise were equally beneficial in counteracting fatigue [9]. This study aimed to evaluate the longer-term effectiveness and cost-effectiveness of HI versus LMI exercise for physical fitness, fatigue, and HRQoL.

## Methods

### Setting and participants

Detailed methods, including sample size calculations, of the REACT study have been reported previously [9, 10]. Briefly, REACT is a multicenter randomized controlled trial (RCT) in cancer patients recruited from nine Dutch hospitals between 2011 and 2013. The Medical Ethics Committee of the VU University Medical Centre approved the study. Patients aged ≥ 18 years with histologically confirmed breast, colon, ovarian, cervix or testis cancer, or lymphomas with no indication of recurrent or progressive disease who had completed (neo-)adjuvant chemotherapy with curative intent were eligible and invited to participate. Exclusion criteria were the following: being unable to perform daily activities; presence of cognitive disorders, severe emotional instability, and diseases that hamper patients’ capacity of carrying out HI exercise; and being unable to read and write Dutch. Written informed consent was obtained from all participants prior to participation.

### Randomization

Following baseline assessments, participants were stratified by cancer type and hospital and randomly assigned to HI exercise, LMI exercise, or WLC using random numbers tables [9]. Shortly after randomization, HI and LMI participants commenced their 12-week exercise program. WLC participants were also randomly allocated to HI or LMI exercise, but started exercising after the 12-week follow-up assessment. Allocation sequence was concealed from the clinical and research staff. Due to the interventions’ nature, participants and physiotherapists were not blinded.

### Exercise interventions

HI and LMI exercise interventions had similar exercise types, durations, and frequencies, but differed in intensity (Table 1). Exercise sessions were given twice per week during 12 weeks and supervised by a trained physiotherapist. Both exercise programs included six resistance exercises targeting large muscle groups (i.e., vertical row, leg press, bench press, pull over, abdominal crunch, and lunge), with a training volume of two sets of ten repetitions [10]. Workload per exercise was defined by an indirect one-repetition maximum (1-RM) measurement. Following a warm-up, the physiotherapist estimated a workload at which the patient was expected to perform four to eight repetitions, taking into consideration age, gender, and height [10]. Furthermore, both programs included two types of endurance interval exercises. During weeks 1–4, patients cycled 2 × 8 min with alternating workloads (defined by the maximum short exercise capacity (MSEC) estimated by the steep ramp test [11]). Patients in the HI exercise group alternated 30 s at 65% of MSEC with 60 s at 30% of MSEC. From the fifth week onwards, the 30 s at 65% of MSEC was alternated with 30 s at 30% of MSEC. The workload for the LMI exercise group alternated between 45 and 30% of MSEC in a similar way. During weeks 5–12, one additional endurance interval session was added substituting one 8-min interval of cycling. This interval session consisted of 3 × 5 min of aerobic exercise at constant workload (defined by the heart rate reserve using the Karvonen formula) [12]. The use of the Karvonen formula allowed patients to perform these aerobic exercises using different ergometers (e.g., cycle ergometer, treadmill). Physiotherapists applied behavioral motivational counseling techniques to overcome possible exercise barriers and to encourage participants to obtain and maintain a physically active lifestyle. At 4, 10, and 18 weeks after intervention completion, three booster sessions (i.e., supervised workout sessions) were provided to motivate participants to maintain their exercise engagements.

**Table 1:** Exercise intensities of the HI and LMI resistance and endurance exercise programs

	Resistance exercises (1-RM)^a^ (6 exercises targeting the large muscle groups^b^, 2 sets of 10 repetitions each)	Endurance interval exercises Part A (MSEC)^a^ (8 min alternating workload)	Endurance interval exercises Part B (HRR)^a^ (3 × 5 min constant workload)	Counseling
High intensity (HI) exercise ^c^	70–85%	65/30%^d^	≥ 80%	Participants were encouraged to start or maintain a physically active lifestyle in addition to the supervised exercise sessions.
Low-to-moderate intensity (LMI) exercise ^c^	40–55%	45/30%^d^	40–50%	

*1-RM*, one-repetition maximum; *MSEC*, maximum short exercise capacity; *HRR*, heart rate reserve;

^a^Every 4 weeks (week 1, 5 and 9), the physiotherapist evaluated training progress and adjusted the workload accordingly

^b^Exercises included vertical row, leg press, bench press, pull over, abdominal crunch, and lunge)

^c^Exercises were accompanied with BORG scores and heart rate monitors to guide the physiotherapists. In the occasion that the training intensity seemed too high or too low, the 1-RM, MSEC, or HRR was reassessed

^d^In the first four weeks, 30 s at 65% of MSEC was alternated with 60 s at 30% for HI, and from the fifth week onwards, intensity was alternated every 30 s. The workload for the LMI exercise group was alternated between 45 and 30% of MSEC in a similar way

### Measurements

Socio-demographic data were collected by self-report. Clinical information was obtained from medical records. Physiotherapists documented session attendance in exercise logs. Outcomes were assessed at baseline, and after 12 and 64 weeks, except for dual energy X-ray absorptiometry (DXA), which was only performed at baseline and 64 weeks. Detailed descriptions of the assessments and their measurement properties are provided elsewhere [10, 13]. Physical tests were performed by an independent assessor.

### Primary outcomes

Cardiorespiratory fitness was measured during a maximal cyclometer exercise test aiming to achieve peak oxygen uptake (peakVO_2_, in mL/kg/min) within 8–12 min [14] following a ramp protocol, in which breath-by-breath gas exchange was measured continuously. After each test, peakVO_2_ (i.e., highest oxygen consumption values averaged over a 15-s interval within the last 60 s), peak power output (peakW, in watt), and the ventilatory threshold (determined by the oxygen equivalent method [14]) were recorded. Hand-grip strength was assessed using a JAMAR hand-grip dynamometer [15] and the mean score (in kg) of three attempts with the participants’ dominant hand was used for further analyses. Lower body function was assessed using the 30-s chair-stand test [16]. The total number of times participants raised to a full stand in 30 s was reported. Fatigue was assessed using the Multidimensional Fatigue Inventory (MFI) [17], including five subscales: general fatigue, physical fatigue, reduced physical activity, reduced motivation, and mental fatigue.

### Secondary outcomes

HRQoL was measured using the European Organisation Research and Treatment of Cancer-Quality of Life questionnaire-Core 30 (EORTC-QLQ-C30) [18] and anxiety and depression by the Hospital Anxiety and Depression Scale (HADS) [19]. Physical activity (PA) was objectively assessed by accelerometers (Actigraph) using vertical accelerations converted into counts/minute. Body mass index (BMI) was calculated from measured body height and weight. Body composition was determined using percentage of total body fat mass (%FM), lean mass (%LM), and lumbar spine (L1–L4) bone mineral density (BMD), measured by DXA with a Hologic Discovery DXA scanner. Quality-adjusted life years (QALYs) were estimated using the EQ-5D-3L [20]. EQ-5D-3L health states were converted into utilities using the Dutch tariff [21]. QALYs were calculated using linear interpolation between measurement points.

### Cost measures

Intervention costs were micro-costed [22, 23]. Attendance of exercise and booster sessions were registered, intervention providers’ time investments were valued using their gross hourly salaries (including overhead), and material costs were estimated using invoices. All other cost categories were assessed using 3-monthly questionnaires, with 3-month recall periods. Healthcare costs included costs due to primary and secondary healthcare use, and medication. Dutch standard costs were used to value healthcare use [23]. Medication use was valued using unit prices of the Royal Dutch Society of Pharmacy [24]. Informal care (i.e., care by family/friends) was valued using a shadow price [23]. Absenteeism was assessed using participants’ reports of their number of absence days and, in case of partial absence, their percentage of normal working hours worked. Using the friction cost approach (FCA), absenteeism costs were valued with age- and gender-specific price weights [23, 25]. The FCA assumes that costs are limited to the friction period (i.e., period needed to replace a sick-listed worker = 23 weeks) [23, 25]. Unpaid productivity (e.g., volunteer work) losses were valued using the aforementioned shadow price [23]. Sports costs included expenses on memberships and equipment. All costs were converted to 2012 euros (€) [26].

### Statistical analyses

Differences in outcomes between HI and LMI exercise interventions at longer-term follow-up were assessed using linear mixed model analyses with a two-level structure (i.e., participants were clustered within hospitals). Both interventions were simultaneously regressed on the longer-term value of the outcome, adjusted for the baseline value, age, gender, and timing of intervention (i.e., direct start or WLC). To check whether missing data affected the results, sensitivity analyses were conducted on an imputed dataset for peakVO_2_, hand-grip strength, and fatigue (SA1). Missing data were multiple imputed using predictive mean matching, stratified by group allocation [27]. The imputation model was specified according to White et al. [27]. Twenty different datasets were created. Pooled estimates were calculated using Rubin’s rules [27].

To evaluate within-group changes in HI and LMI exercise interventions from short-term to longer-term follow-up, we conducted linear mixed models for repeated measurements (i.e., repeated measurements were clustered within patients, which were clustered within hospitals). This model simultaneously regressed the intervention effect on short term and longer term and included time and the interaction between time and exercise group as determinants and age, gender, and the outcome’s baseline value as covariates.

Cost-effectiveness analyses were performed from the societal perspective using the multiple imputed datasets [27]. Between-group differences were estimated for total and disaggregated costs. Total cost and effect differences were estimated using linear mixed model analyses, adjusted for baseline, age, gender, and intervention timing. Incremental cost-effectiveness ratios (ICERs) were calculated by dividing the adjusted total cost differences by those in effects [6]. Uncertainty around cost differences and ICERs was estimated using bias-corrected (BC) bootstrap intervals (5000 replications, stratified by hospital) [28]. Cost-effectiveness planes [29] and cost-effectiveness acceptability (CEA) curves were constructed [30]. A post hoc analysis was performed applying a healthcare perspective and a sensitivity analysis (SA2) was conducted assuming that all scheduled exercise sessions needed to be paid for, rather than only those attended.

As disease recurrence—which may influenced quality of life and healthcare costs—occurred more often during follow-up in LMI exercise, additional sensitivity analyses (SA3–4) were performed. We excluded patients with disease recurrence (*n* = 17) in order to check whether disease recurrence affected the results of the main effectiveness (i.e., between-group difference—SA3) and cost-effectiveness analyses (SA4).

All primary analyses were performed according to intention-to-treat. Costs and effects beyond 1 year were discounted at a rate of 4 and 1.5%, respectively [23]. Effectiveness analyses were performed in SPSS (v22.0) and multiple imputation and cost-effectiveness analyses in STATA (v12.0). *P* < 0.05 was considered significant.

#### Data availability

At present, raw data of the REACT study forms part of a PhD project, including current data on long-term (cost-)effectiveness. As a consequence, currently, we are unable to publish this dataset. However, the REACT dataset is included in the internationally shared POLARIS database [31], and researchers who are interested to collaborate are invited to prepare a paper proposal.

## Results

**Fig. 1:**
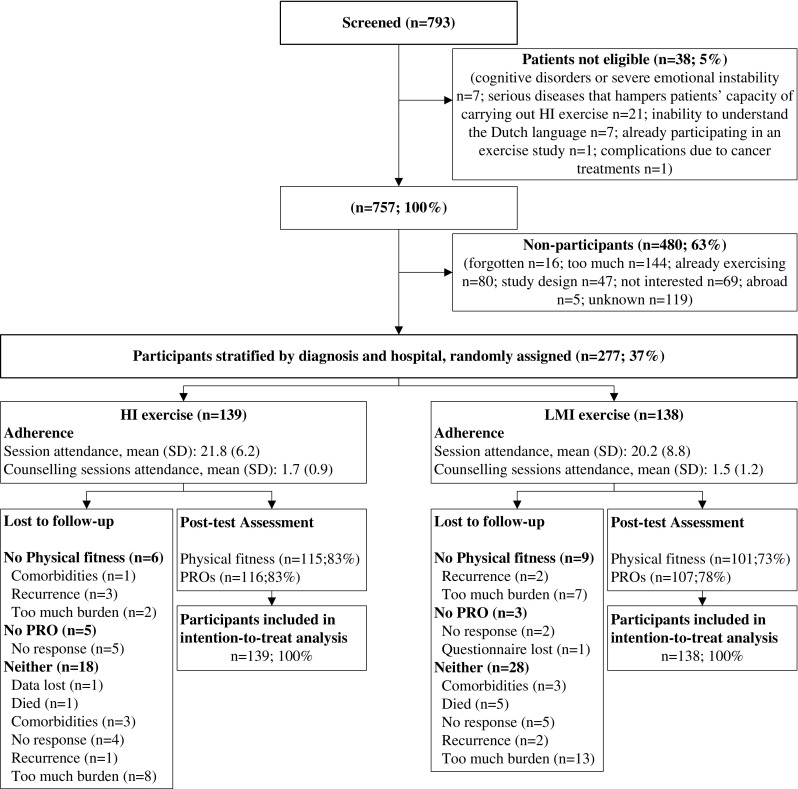
Patients flowchart of the REACT study. *HI*, high intensity exercise; *LMI*, low-to-moderate intensity exercise; *WLC*, wait list control group; *PRO*, patient-reported outcomes

Of the 757 eligible patients, 277 (37%) participated (Fig. 1). Age, gender, and cancer type did not differ significantly between participants and non-participants [9]. The participants’ baseline characteristics were balanced across groups (Table 2). On average, participants in HI and LMI groups attended 20.2 (SD = 8.8) and 21.8 (SD = 6.2) of 24 exercise sessions and 1.5 (SD = 1.2) and 1.7 (SD = 0.9) of 3 booster sessions, respectively (Fig. 1). There were no adverse events directly related to the interventions. Complete physical fitness and patient-reported outcome data were obtained from 116 (80%) and 223 (81%) participants, respectively. Furthermore, 211 (76%), 185 (66%), 179 (65%), 173 (63%), and 176 (64%) participants had complete cost data at 3, 6, 9, 12, and 15 months, respectively.

**Table 2:** Baseline characteristics of the participants

Characteristics	LMI *n* = 138	HI *n* = 139
Socio-demographic		
Age, mean (SD) (years)	53 (11.4)	54 (10.7)
Gender, *n* (%) male	26 (19)	29 (21)
Partner, *n* (%) yes	120 (87)	112 (81)
Education, *n* (%)^a^		
Low	19 (14)	28 (20)
Intermediate	64 (47)	58 (42)
High	53 (39)	52 (38)
Being employed, *n* (%)		
Employed	30 (22)	36 (26)
Not employed	82 (59)	85 (61)
Retired	26 (19)	18 (13)
Smoking, *n* (%) yes^b^	8 (6)	9 (7)
Comorbidities ≥ 2, *n* (%) yes	14 (10)	16 (12)
Sport history, *n* (%) yes^c^	83 (61)	72 (52)
Exercise during chemotherapy, *n* (%) yes^a^	25 (18)	27 (20)
Clinical		
Cancer type, *n* (%)		
Breast	89 (65)	92 (66)
Colon	24 (17)	25 (18)
Ovarian	4 (3)	8 (6)
Lymphoma	16 (12)	10 (7)
Cervix	4 (3)	0
Testis	1 (1)	4 (3)
Cancer stage, *n* (%)		
Local	84 (61)	103 (74)
Advanced	54 (39)	36 (26)
Type of treatment, *n* (%) yes		
Surgery	123 (89)	127 (91)
Radiation therapy	61 (44)	74 (53)
Surgery + radiation therapy	58 (42)	68 (49)
Immunotherapy	36 (26)	23 (17)
Hormonal therapy	61 (44)	67 (48)
Type of chemotherapy, *n* (%)		
TAC	47 (34)	56 (40)
FEC	9 (7)	10 (7)
TAC/FEC combinations	30 (22)	23 (17)
Capecitabine and oxaliplatin	14 (10)	12 (9)
Oxaliplatin combinations	10 (7)	12 (9)
Carboplatin and paclitaxel	8 (6)	10 (7)
CHOP	11 (8)	7 (5)
ABVD	4 (3)	4 (3)
Cisplantin	3 (2)	0
BEP	1 (1)	3 (2)
Other	1 (1)	2 (1)

*n* number; *FEC* fluorouracil, epirubicin, and cyclophosphamide; *TAC* taxotere, adriamycin, and cyclophosphamide; *CHOP* cyclophosphamide, doxorubicin, vincristine, and prednisone; *ABVD* doxorubicin, bleomycin, vinblastine, and dacarbazine; *BEP* bleomycin, etoposide, and cisplatin

^a^*n* − 3

^b^*n* − 4

^c^*n* − 1

At longer term, intervention effects on role functioning (*β*_between-group difference_ = 5.9, 95% CI = 0.5; 11.3) and social functioning (*β*_between-group difference_ = 5.7, 95% CI = 1.7; 9.6) were larger for HI than for LMI exercise (Table 3). No other significant between-group differences were found at longer term. Results of the sensitivity analyses (SA3) were comparable (data not shown).

**Table 3:** Mean (SD) values at baseline and follow-up and differences in effects on primary and secondary outcomes between groups (adjusted model, corrected for age and gender)

	LMI (*n* = 138)	64 weeks follow-up	HI (*n* = 139)	64 weeks follow-up	HI vs. LMI	HI vs. LMI^a^	*∆*Time LMI	∆Time HI
	Baseline		Baseline		*β* (95% CI)	*β* (95% CI)	*β* (95% CI)	*β* (95% CI)
	Mean (SD)	Mean (SD)	Mean (SD)	Mean (SD)				
Cardiorespiratory fitness^b^								
PeakVO2 (mL/kg/min)	22.1 (5.8)	25.6 (6.8)	22.0 (6.5)	26.3 (8.1)	0.7 (− 0.3; 1.7)	0.5 (− 0.9; 1.9)	0.1 (− 0.8; 0.9)	− 0.5 (− 1.3; 0.3)
WMax (W)	136 (43)	155 (48)	137 (45)	162 (55)	6.4 (0.6; 12.3)*		2.2 (− 1.6; 5.9)	1.8 (− 1.8; 5.4)
Anaerobic threshold (mL/kg/min)	16.1 (4.6)	18.0 (5.2)	15.9 (4.9)	18.5 (5.7)	0.6 (− 0.4; 1.6)		− 0.7 (− 1.6; 0.1)	− 1.1 (− 1.9; − 0.2)*
Muscle strength								
Sit to stand (stands)^c^	16 (3.7)	20 (5.2)	17 (4.2)	20 (5.2)	− 0.4 (− 1.4; 0.5)		1.2 (0.5; 1.9)*	1.4 (0.7; 2.2)*
Hand-grip strength (kg)^d^	33.2 (9.5)	35.9 (11.0)	32.8 (10.0)	35.6 (11.4)	− 0.4 (− 1.6; 0.8)	− 0.2 (− 2.1; 1.8)	0.1 (− 0.7; 0.9)	1.4 (0.6; 2.2)*
Fatigue (range 1–20)^e^								
General fatigue^f^	12.9 (4.2)	11.7 (1.3)	12.7 (3.8)	11.7 (1.6)	− 0.1 (− 0.4; 0.3)	− 0.2 (− 0.6; 0.3)	1.5 (0.7; 2.3)*	2.0 (1.2; 2.6)*
Physical fatigue^f^	12.8 (4.0)	13.0 (1.4)	12.9 (3.9)	12.8 (1.6)	− 0.2 (− 0.6; 0.1)		3.8 (3.0; 4.6)*	4.1 (3.3; 4.8)*
Reduced activity^g^	11.7 (3.5)	12.5 (1.2)	12.0 (3.5)	12.4 (1.5)	− 0.1 (− 0.5; 0.3)		3.5 (2.7; 4.2)*	3.0 (2.3; 3.7)*
Reduced motivation^h^	8.7 (3.1)	12.1 (1.7)	9.0 (3.0)	12.1 (1.8)	0.02 (− 0.4; 0.5)		3.7 (3.0; 4.4)*	4.6 (3.9; 5.2)*
Mental fatigue^f^	10.8 (4.1)	11.8 (1.1)	11.0 (4.0)	11.7 (1.4)	− 0.03 (− 0.3; 0.3)		1.8 (1.0; 2.6)*	2.1 (1.3; 2.9)*
Health-related quality of life (range 0–100)^i^								
Global QoL	73.2 (16.7)	80.0 (16.5)	71.3 (15.8)	83.0 (15.6)	3.7 (− 0.3; 7.7)^†^		0.7 (− 2.7; 4.0)	0.4 (− 2.9; 3.7)
Physical functioning	82.1 (12.9)	87.6 (14.8)	80.4 (15.3)	89.7 (11.9)	2.9 (− 0.1; 5.9)^†^		− 0.4 (− 2.8; 1.9)	2.2 (− 0.1; 4.5)^†^
Role functioning	70.9 (25.1)	83.5 (24.5)	68.5 (26.7)	88.8 (19.4)	5.9 (0.5; 11.3)*		1.1 (− 4.2; 6.3)	5.5 (0.3; 10.6)*
Emotional functioning	83.5 (16.3)	85.3 (18.1)	85.4 (16.5)	87.4 (17.4)	0.9 (− 2.8; 4.6)		1.0 (− 2.4; 4.3)	− 0.8 (− 4.1; 2.4)
Cognitive functioning	77.7 (23.0)	83.8 (17.9)	79.5 (21.6)	83.9 (21.2)	− 0.7 (− 4.8; 3.4)		5.7 (2.0; 9.4)*	2.1 (− 1.6; 5.7)
Social functioning	78.8 (21.3)	87.2 (19.0)	76.7 (24.1)	92.2 (15.5)	5.7 (1.7; 9.6)*		1.1 (− 2.6; 4.8)	− 0.5 (− 1.3; 0.3)
Distress (range 0–21)^j^								
Anxiety^h^	3.9 (2.8)	3.9 (3.1)	3.8 (3.0)	3.9 (3.5)	0.2 (− 0.4; 0.9)		− 0.1 (− 0.7; 0.6)	0.7 (0.1; 1.3)*
Depression^k^	3.1 (2.8)	2.8 (3.3)	3.2 (2.7)	2.6 (3.0)	− 0.3 (− 0.9; 0.4)		0.1 (− 0.5; 0.6)	0.1 (− 0.4; 0.7)
Body composition								
BMI (kg/m^2^)	26.7 (4.3)	26.9 (4.5)	26.9 (4.5)	27.0 (4.6)	− 0.02 (− 0.4; 0.4)		0.1 (− 0.2; 0.3)	0.3 (0.03; 0.5)*
Percentage fat mass^l^	31.7 (7.4)	33.5 (7.4)	32.1 (6.9)	33.1 (8.3)	− 0.7 (− 1.7; 0.3)			
Percentage lean mass	64.6 (7.5)	63.5 (7.0)	64.9 (6.5)	63.3 (9.2)	− 0.4 (− 1.7; 0.9)			
BMD lumbar spine (g/cm^2^)^m^	1.0 (0.2)	1.0 (0.2)	1.0 (0.2)	1.0 (0.2)	− 0.01 (− 0.02; 0.01)			
Physical activity								
Accelerometer (CPM)^n,o^	254.0 (96.1)	243.4 (165.4)	243.7 (100.4)	217.9 (139.9)	− 22.4 (− 65.3; 20.5)		− 21.9 (− 61.8; 17.9)	− 26.3 (− 65.6; 13.0)

*LMI*, low-to-moderate-intensity exercise; *HI*, high-intensity exercise; *SD*, standard deviation; *n*, number; *kg*, kilogram; *W*, watt; *BMI*, body mass index; *BMD*, bone mineral density; *CPM*, counts per minute

*p < 0.05); ^†^0.05 ≤ *p* < 0.10

^a^Sensitivity analysis imputed dataset

^b^Missings due to technical problems (*n* = 2), or discomfort (*n* = 1)

^c^Missings due to musculoskeletal problems (*n* = 3)

^d^Missings due to musculoskeletal problems (*n* = 11)

^e^Higher score means a higher level of self-reported fatigue in all subscales

^f^Missing due to incomplete questionnaire (*n* = 2)

^g^Missing due to incomplete questionnaire (*n* = 1)

^h^Missing due to incomplete questionnaire (*n* = 3)

^i^Higher score means a higher level of self-reported HRQoL in all subscales

^j^Higher score means a higher level of anxiety and depression in both subscales

^k^Missing due to incomplete questionnaire (*n* = 8)

^l^Missings due to no show (*n* = 2)

^m^Missings due to no show (*n* = 2) or technical problems (*n* = 2)

^n^Average counts for *Y*-axis

^o^Missings due to technical problems/insufficient wearing-time (*n* = 37)

No significant within-group changes were found for peakVO_2_ and HRQoL between short and longer terms for both HI and LMI exercise interventions, indicating that the intervention-induced improvements at short term were maintained at longer term (Table 3). For HI exercise, role functioning (*β*_within-group change_ = 5.5, 95% CI = 0.3; 10.6), hand-grip strength (*β*_within-group change_ = 1.4, 95% CI = 0.6; 2.2), and BMI (*β*_within-group change_ = 0.3, 95% CI = 0.03; 0.5) increased from short to longer term, and lower body muscle function increased both in HI and LMI exercise interventions (HI—*β*_within-group change_ = 1.4, 95% CI = 0.7; 2.2, LMI—*β*_within-group change_ = 1.2, 95% CI = 0.5; 1.9). For both groups, significant within-group changes were found for fatigue and anxiety, such that they returned to baseline levels. No significant within-group changes from short to longer term were found for depression and objectively measured PA.

Total societal costs did not differ significantly between HI and LMI exercise interventions (*β* = − 2429€, 95% CI = − 5798; 933). In HI exercise, healthcare costs were significantly lower (*β* = − 2056€, 95% CI = − 3816; − 443) and intervention costs were significantly higher (*β* = 40€, 95% CI = 8; 75) than in LMI exercise (Table 4). For QALYs, an ICER of − 87,831 was found, indicating that HI exercise was associated with a cost saving of 87,831€/QALY gained, compared with LMI exercise (Table 5). When societal decision-makers are not willing to pay anything per unit of effect gained, the probability of HI exercise being cost-effective compared with LMI exercise was 0.87. This probability increased to 0.91 at a willingness-to-pay of 20,000€/QALY and reaching 0.95 at 52,000€/QALY. For hand-grip strength, the probability of cost-effectiveness increased as the willingness-to-pay increased, from 0.87 to 0.95 at 58.000€/kg, while it decreased for peakVO_2_ and general fatigue (data not shown). From a healthcare perspective, results were more favorable for HI exercise as shown by higher probabilities of cost-effectiveness, e.g., if healthcare decision-makers are not willing to pay anything per unit of effect gained, the probability of cost-effectiveness was 0.97 for all outcome measures. When we assumed that all scheduled exercise sessions needed to be paid for SA2, we found comparable results. When patients who had a disease recurrence during follow-up were excluded from the analyses (SA4), the mean difference in total societal costs between HI and LMI exercise interventions was smaller (i.e., − 1366€ versus − 2429€). Additionally, HI exercise had slightly lower probabilities of being cost-effective in comparison with LMI exercise (i.e., 0.89 versus 0.96 at 80,000€/QALY). However, the societal cost difference was in favor of HI exercise, in both the main analysis and SA4, and the differences in effect were comparable.

**Table 4:** Mean costs per participant in the high intensity (HI) and low-to-moderate intensity (LMI) exercise groups and cost differences between both groups during follow-up

Cost category	LMI *n* = 138; mean (SEM)	HI *n* = 139; mean (SEM)	Mean cost difference Model 1^a^ (95% CI)	Mean cost difference Model 2^b^ (95% CI)	Mean cost difference Model 3^c^ (95% CI)
Intervention costs (€)	815 (15)	858 (11)	43 (8; 77)	40 (7; 76)	42 (8; 75)
Healthcare costs (€)	6232 (993)	4148 (522)	− 2075 (− 3816; − 464)	− 2043 (− 3851; − 438)	− 2056 (− 3816; − 443)
Primary care	2494 (385)	2127 (384)	− 370 (− 1102; 471)	− 333 (− 1073; 491)	− 342 (− 1056; 493)
Secondary care	2644 (657)	1515 (226)	− 1121 (− 2237; − 204)	− 1131 (− 2295; − 201)	− 1134 (− 2274; − 200)
Medication	1093 (227)	505 (75)	− 584 (− 917; − 280)	− 578 (− 915; − 276)	− 584 (− 917; − 268)
Informal care costs (€)	1964 (344)	2095 (478)	136 (− 590; 949)	163 (− 566; 969)	151 (− 552; 954)
Absenteeism costs (€)	7527 (942)	6759 (845)	− 696 (− 2630; 1241)	− 523 (− 2462; 1369)	− 523 (− 2450; 1394)
Unpaid productivity costs (€)	264 (35)	197 (29)	− 67 (− 140; 6)	− 63 (− 137; 8)	− 67 (− 138; 5)
Sports costs (€)	552 (73)	566 (90)	18 (− 138; 192)	25 (− 132; 197)	26 (− 128; 197)
Total costs (€)	17,355 (1720)	14,623 (1327)	− 2641 (− 5983; 767)	− 2400 (− 5850; 942)	− 2429 (− 5798; 933)

*€*, euro; *n*, number; *CI*, confidence interval; *SEM*, standard error of the mean

^a^Solely corrected for follow-up duration

^b^Corrected for follow-up duration, age, and gender

^c^Random intercept for hospital and corrected for follow-up duration, age, and gender

**Table 5:** Differences in pooled mean costs and effects (95% confidence intervals), incremental cost-effectiveness ratios, and the distribution of incremental cost-effect pairs around the quadrants of the cost-effectiveness planes

Analysis	Sample size		Outcome	∆*C* (95% CI)	∆*E* (95% CI)	ICER	Distribution CE-plane (%)			
	LMI	HI		€	Points	€/point	NE^1^	SE^2^	SW^3^	NW^4^
Main analysis—imputed dataset	138	139	QALYs (range 0–1)	− 2429 (− 5798; 933)	0.028 (− 0.006; 0.061)	− 87,831	13.2	55.3	17.7	13.8
	138	139	General fatigue (0–20)	− 2429 (− 5798; 933)	− 0.16 (− 0.61; 0.29)	15,116	10.3	65.6	22.2	1.7
	138	139	Grip strength (kg)	− 2429 (− 5798; 933)	0.14 (− 1.72; 2.01)	1411	7.6	50.3	37.6	4.5
	138	139	PeakVO_2_ (mL/kg/min)	− 2429 (− 5798; 933)	− 0.02 (− 1.40; 1.37)	159,236	5.1	42.7	45.3	7.0
Post hoc analysis—healthcare perspective	138	139	QALYs (range 0–1)	− 2015 (− 3786; − 412)	0.028 (− 0.006; 0.061)	− 72,859	1.2	93.1	5.5	0.2
	138	139	General fatigue (0–20)	− 2015 (− 3786; − 412)	− 0.16 (− 0.61; 0.29)	12,540	1.1	74.8	23.9	0.2
	138	139	Grip strength (kg)	− 2015 (− 3786; − 412)	0.14 (− 1.72; 2.01)	− 14,096	0.7	57.1	41.5	0.6
	138	139	PeakVO_2_ (mL/kg/min)	− 2015 (− 3786; − 412)	− 0.02 (− 1.40; 1.37)	132,093	0.6	47.1	51.5	0.7
Sensitivity analysis—fixed intervention costs	138	139	QALYs (range 0–1)	− 2471 (− 5849; 907)	0.028 (− 0.006; 0.061)	− 89,341	10.6	83.7	4.6	1.1
	138	139	General fatigue (0–20)	− 2471 (− 5849; 907)	− 0.16 (− 0.61; 0.29)	15,376	10.0	65.9	22.3	1.7
	138	139	Grip strength (kg)	− 2471 (− 5849; 907)	0.14 (− 1.72; 2.01)	− 17,285	7.4	50.5	37.8	4.4
	138	139	PeakVO_2_ (mL/kg/min)	− 2471 (− 5849; 907)	− 0.02 (− 1.40; 1.37)	161,974	5.0	42.8	45.5	6.8
Sensitivity analysis—patients with disease recurrence excluded (SA4)	126	134	QALYs (range 0–1)	− 1366 (− 4692; 2063)	0.025 (− 0.009; 0.059)	− 54,228	21.5	70.6	4.5	3.4
	126	134	General fatigue (0–20)	− 1366 (− 4692; 2063)	− 0.18 (− 0.63; 0.26)	7389	21.2	58.7	16.4	3.7
	126	134	Grip strength (kg)	− 1366 (− 4692; 2063)	0.16 (− 1.58; 1.90)	− 8517	14.2	43.0	32.1	10.7
	126	134	PeakVO_2_ (mL/kg/min)	− 1366 (− 4692; 2063)	− 0.03 (− 1.31; 1.25)	48,349	11.8	37.1	38.0	13.2

*C*, costs; *E*, effects; *ICER*, incremental cost-effectiveness ratio; *CE-plane*, cost-effectiveness-plane; *QALYs*, quality-adjusted life years

^1^Refers to the northeast quadrant of the CE-plane, indicating that high-intensity training is more effective and more costly than low-to-moderate-intensity training

^2^Refers to the southeast quadrant of the CE-plane, indicating that high-intensity training is more effective and less costly than low-to-moderate-intensity training

^3^Refers to the southwest quadrant of the CE-plane, indicating that high-intensity training is less effective and less costly than low-to-moderate-intensity training

^4^Refers to the northwest quadrant of the CE-plane, indicating that high-intensity training is less effective and more costly than low-to-moderate-intensity training

## Discussion

At longer term (i.e., 64 weeks), effects on role and social functioning were significantly larger for HI than for LMI exercise. Within-group changes showed that intervention-induced improvements in cardiorespiratory fitness and HRQoL found at short term were successfully maintained at longer term for HI and LMI exercise interventions, whereas fatigue returned to baseline levels. Also, HI exercise was cost-effective for QALYs, compared to LMI exercise.

The mean improvements in peakVO_2_ after exercise (HI 4.3 mL/kg/min, LMI 3.5 mL/kg/min) at longer term were in line with the mean improvement of 3.3 mL/kg/min found in cancer survivors after supervised exercise, as reported in a previous meta-analysis [2]. In contrast with the tendency of a dose-response relationship of exercise intensity at short term [9], no significant differences in peakVO_2_ were found at longer term between HI and LMI exercise. Nevertheless, the exercise-induced benefits on peakVO_2_ at short term were successfully maintained over time in both exercise groups. Although this is hopeful, we should acknowledge that, compared to healthy adults, the patients’ level of peakVO_2_ at longer term was still “poor” [14]. Apparently, a 12-week exercise program is too short for patients to fully recover to normative values. On the other hand, there may be a possibility that patients do not fully recover after having received multiple cancer treatments.

At longer term, hand-grip strength and lower body muscle function were not significantly different between HI and LMI exercise interventions. A previous meta-regression analysis revealed that the effects of resistance training on muscle strength may be more dependent on volume than on intensity [32]. Additional head-to-head comparisons of exercise programs with different exercise parameters (i.e., frequency, intensity, type, time) are therefore warranted to define the optimal exercise dose on muscle strength for cancer survivors. Furthermore, increases in both strength outcomes between short and longer terms for both groups suggest that these improvements result from increased uptake of daily activities during follow-up.

At longer term, self-reported fatigue did not differ significantly between HI and LMI exercise interventions, and in both groups, it returned to baseline values between short and longer terms. This lack of sustainable improvements in fatigue is in line with previous studies [3] and may be related to the patients’ low self-efficacy in managing fatigue, particularly while resuming daily activities without supervision and support from a physiotherapist [33]. On the other hand, self-reported fatigue in a longitudinal study is also susceptible to “response-shift bias,” resulting from a change in the internal standard of fatigue perception throughout the cancer continuum [34].

We found a significant better social and role functioning for HI exercise compared to LMI exercise at longer term. In addition, longer-term effects on global QoL and physical functioning tended to be larger for HI than for LMI exercise, but this was not significant. Overall, current findings reveal a possible dose-response relationship of exercise intensity for some HRQoL domains among cancer survivors. Hence, a previous meta-analysis reported significant exercise effects on global QoL and social functioning, but not on role functioning [4]. Based on our significant effects on role functioning, it may be hypothesized that participants gain confidence from completing a HI exercise program [35], resulting in improvements in a person’s role in society. Furthermore, the exercise-induced benefits on HRQoL were successfully maintained over time in both interventions, despite the return to baseline levels of fatigue. This indicates that besides fatigue, which is found to mediate the exercise effect on HRQoL [36], other factors also contribute to HRQoL.

The lack of a significant difference between HI and LMI exercise interventions in psychological distress at longer term is in contrast with a previous meta-analysis reporting small but significant reductions in depression and anxiety after exercise at short and longer terms, compared to usual care [4, 37]. Yet, our study lacked a non-exercise group and the mean values for both outcomes were already low at baseline, leaving little room for improvement. Furthermore, from short to longer term, anxiety returned to baseline in both groups, despite the beneficial short-term intervention effects on anxiety after HI exercise [9]. So, HI exercise might be more effective in reducing anxiety compared to LMI exercise; however, sustainability is lacking which may reflect the vulnerability of psychosocial recovery [38].

Comparable with our short-term findings [9], there were no significant differences between HI and LMI exercise interventions in body composition and objectively measured PA at the longer term. This may be related to the design of our exercise interventions. To successfully reduce fat mass, complementary dietary changes may be required [39] and improving and maintaining PA may require specific behavioral change techniques (e.g., motivational interviewing [40], goal setting [41]). Our finding that BMI significantly increased from short to longer term in HI exercise is unexpected, and its clinical meaningfulness may be questioned, as it was not supported by changes in %FM and %LM.

At the lower bounds of the Dutch and UK willingness-to-pay threshold (i.e., 20,000 and 24,400€/QALY gained, respectively), the probability of HI exercise being cost-effective compared to that of LMI exercise was ≥ 0.91 and increased even more with increasing willingness-to-pay values. Thus, at longer term, HI exercise can be considered cost-effective compared with LMI exercise for QALYs, if decision-makers are willing to accept a probability of cost-effectiveness of 0.91 and to pay 20,000€/QALY. The relatively high probabilities of cost-effectiveness seemed to be related to lower healthcare costs in HI exercise. Although smaller, the healthcare costs were still lower after excluding patients with disease recurrence, and HI exercise remained cost-effective. Current results support previous results of a systematic review showing acceptable cost-effectiveness ratios for cancer rehabilitation programs that produced significant health gains [8] compared to usual care. As willingness-to-pay thresholds are lacking for peakVO_2_, hand-grip strength, and general fatigue, strong conclusions about HI’s cost-effectiveness as compared to LMI exercise for these outcomes cannot be made.

Strengths of this study include the direct comparison between HI and LMI exercise interventions, longer-term effectiveness and cost-effectiveness analyses, multicentre RCT design, large sample size, the use of valid and reliable outcome measures, and the use of state-of-the-art statistical methods. However, some limitations are noteworthy. First, to limit non-participation and minimize contamination, a WLC was included instead of a non-exercising control group. Therefore, at longer term, we were only able to evaluate the effectiveness and cost-effectiveness of HI compared to LMI exercise, because all participants had received an exercise intervention at 64 weeks. Second, cost data were collected using self-report, which may have caused “social desirability” and/or “recall bias.” Third, a relatively large number of participants had missing cost data. To deal with this limitation, missing data were multiple imputed [42]. Finally, it should be acknowledged the CEA results might not be generalized to other countries with different healthcare systems and/or payment structures [43].

## Conclusions

In conclusion, at longer-term follow-up, we found a larger intervention effect on role and social functioning for HI than for LMI exercise. Exercise-induced benefits in peakVO_2_ and HRQoL were successfully maintained between short and longer terms, but not for fatigue. Furthermore, HI exercise was cost-effective for QALYs compared to LMI exercise, mostly due to significant lower healthcare costs in HI exercise. Hence, the current findings advocate the implementation of supervised exercise as part of standard cancer care, and if possible HI exercise.

## Abbreviations

1-RM: one-repetition maximum
%FM: percentage of total body fat mass
%LM: percentage of total lean mass
BC: bias-corrected
BMD: bone mineral density
BMI: body mass index
CEA: cost-effectiveness acceptability
DXA: dual energy X-ray absorptiometry
EORTC-QLQ-C30: European Organisation Research and Treatment of Cancer-Quality of Life questionnaire-Core 30
FCA: friction cost approach
HADS: Hospital Anxiety and Depression Scale
HI: high intensity
HRQoL: health-related quality of life
HRR: heart rate reserve
ICERs: incremental cost-effectiveness ratios
LMI: low-to-moderate intensity
MFI: Multidimensional Fatigue Inventory
MSEC: maximum short exercise capacity
PA: physical activity
PeakVO_2_: peak oxygen uptake
PeakW: peak power output
QALYs: quality-adjusted life years
QoL: quality of life
REACT: Resistance and Endurance exercise After ChemoTherapy
RCT: randomized controlled trial
SA: sensitivity analyses
WLC: wait list control
